# Long‐Pulse Endoscopic Laser Lithotripsy for Right Intrahepatic Bile Duct Stones via an Endoscopic Ultrasound–Guided Created Route

**DOI:** 10.1111/den.70264

**Published:** 2026-08-20

**Authors:** Ritsuko Oishi, Haruo Miwa, Shin Maeda

**Affiliations:** ^1^ Gastroenterological Center Yokohama City University Medical Center Yokohama Japan; ^2^ Department of Gastroenterology Yokohama City University Graduate School of Medicine Yokohama Japan

**Keywords:** intrahepatic bile duct stones, laser lithotripsy, long‐pulse mode

Removal of intrahepatic bile duct (IHBD) stones via endoscopic ultrasound–guided created route (ESCR) has been reported as an effective treatment option in patients with surgically altered anatomy [1, 2]. However, extraction of stones located in the right IHBD remains technically challenging. Endoscopic laser lithotripsy (LL) using a Holmium:YAG laser (LithoEVO; EDAP TMS, Lyon, France) can be performed in either short‐ or long‐pulse modes. In particular, the long‐pulse mode delivers laser energy over a longer duration, resulting in finer stone fragmentation [3]. This approach may reduce the need for technically demanding stone extraction maneuvers and facilitate spontaneous clearance of stone fragments through the hepaticojejunostomy anastomosis (Figure 1). Herein, we report complete removal of right IHBD stones using long‐pulse LL via ESCR (Video 1).

**FIGURE 1:**
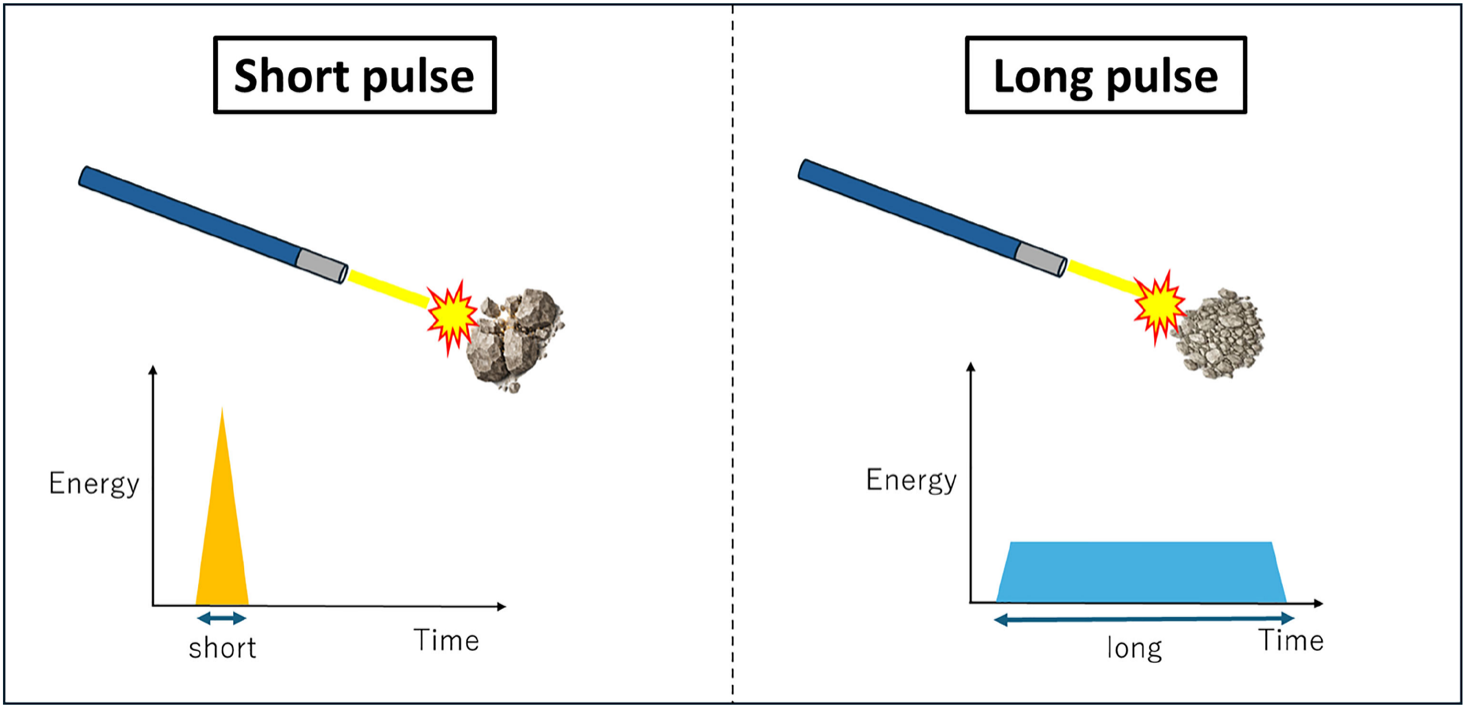
Endoscopic laser lithotripsy can be performed using short‐ or long‐pulse modes. The long‐pulse mode delivers laser energy over a longer duration, resulting in finer stone fragmentation.

**VIDEO 1:** Long‐pulse laser lithotripsy of right intrahepatic bile duct stones through the endosonographically created route after EUS‐guided hepaticogastrostomy. Video content can be viewed at https://onlinelibrary.wiley.com/doi/10.1111/den.70264.

A 78‐year‐old man who had undergone surgery for gastric cancer with Billroth II reconstruction and choledochojejunostomy was admitted for IHBD stone removal because of recurrent cholangitis. Although single‐balloon enteroscope‐assisted endoscopic retrograde cholangiography was attempted, the mechanical lithotripter could not be advanced because of acute angulation of the right IHBD. Endoscopic ultrasound‐guided hepaticogastrostomy was then performed. One month later, LL was performed via ESCR using a slim cholangioscope (eyeMax 9‐Fr; Micro‐Tech, Nanjing, China). The long‐pulse mode maintained a stable cholangioscopic view without stone retropulsion, resulting in fine fragmentation (Figure 2). At a second session performed 2 weeks later, cholangiography showed that most right IHBD stones had disappeared, suggesting effective fragmentation by long‐pulse LL. The remaining small fragments were removed using a balloon catheter.

**FIGURE 2:**
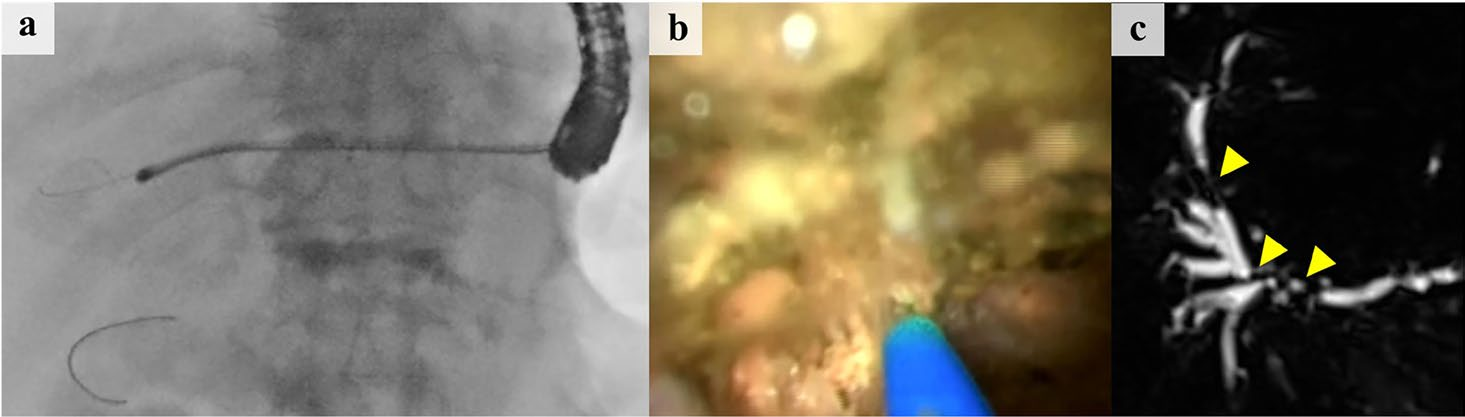
(a) Fluoroscopic image shows a slim cholangioscope advanced into the right intrahepatic bile duct via the endoscopic ultrasound–guided created route. (b) The cholangioscopic view during long‐pulse laser lithotripsy shows fine stone fragmentation. (c) Magnetic resonance cholangiopancreatography shows multiple stones in the right intrahepatic bile duct (yellow arrowheads).

Previous studies have demonstrated the feasibility of LL for difficult biliary stones and antegrade LL via ESCR [4, 5]. To the best of our knowledge, this is the first report of complete removal of right IHBD stones using long‐pulse LL via ESCR. The long‐pulse mode facilitates effective stone clearance even in a nondilated and anatomically challenging bile duct.

## Author Contributions

Haruo Miwa supervised this procedure, and Shin Maeda supervised writing this article.

## Funding

The authors have nothing to report.

## Conflicts of Interest

The authors declare no conflicts of interest.

## Data Availability

The data that support the findings of this study are available on request from the corresponding author. The data are not publicly available due to privacy or ethical restrictions.
